# The Nature and Treatment of Cancer

**Published:** 1846-01

**Authors:** 


### Art. XX.

*The Nature and Treatment of Cancer.* By W. H. Walshe, m.d., Professor of Pathological Anatomy in University College, Physician to University College Hospital, and to the Hospital for Consumption and Diseases of the Chest.—*London,* 1845. 8vo, pp. 590.

This work has reached us at so late a period of the quarter, as puts it out of our power to lay before our readers any such account of it as its importance demands. We have, however, seen enough in the imperfect examination we have been able to make, to convince us, that the volume before us constitutes one of the completest and most valuable monographs of an individual disease that exists in medical literature. In our next Number we hope to prove the correctness of this judgment by an ample analysis of its contents. We are induced to notice the volume on the present occasion, chiefly that we may make our readers acquainted with the improved method of treating cancer, introduced into practice a few years since by Dr. Arnott,—and which is here fully set forth by Dr. Walshe for the first time. We have been constantly expecting that this great invention—for it is really such—would have been given to the world, formally and in detail, by its author; but Dr. Arnott would seem to be as careless of ordinary fame, as he manifestly is of what is as generally prized in these later days—money. He has successively given to the world—truly *given,* given unconditionally and without any hope or possibility of pecuniary return—invention after invention, all designed for, and ministering, in the most striking manner to, the improvement of human health and comfort, and the relief of human suffering; which, if he had been so minded, might have become to him the certain means of almost boundless wealth. First, the hydrostatic or water-bed, and strap-hammock bed, for invalids; then the self-regulating air and water stoves known by his name; then the air-pump, the self-poised chimney-valve, and fish-gill air-warmer, for heating and ventilating ships, public buildings, and rooms; and, lastly, this philosophical apparatus for supplying pressure in morbid growths: a series of effective appliances for the promotion of human weal which, we venture to say, will not merely transmit Dr. Arnott's name to future times as one of the most inventive geniuses and enlightened philanthropists, but will even form no mean element in constituting the present time an epoch in the glorious history of human amelioration. Had NEIL ARNOTT lived in the later days of classical antiquity, he would have had statues raised to him in his lifetime in the public places of his country; had he flourished in a yet earlier age, he would have come down to posterity among those benefactors of their race, whom the simple and beautiful faith of Humanity's childhood has gratefully enrolled among superior beings.

Dr. Walshe's volume is divided into two parts: "The first is devoted to the subject of Cancer in general; the second to the description of the disease as it occurs in all those tissues and organs of the body in which experience has established the fact of its existence." In both of these departments, we believe the reader will not be disappointed if he expects to find everything that was already known on the subject of cancer placed in the clearest possible light, and not a little also that is novel both in fact and doctrine.

We must here also refer, in passing, to another thing that has particularly struck us in going over Dr. Walshe's book—its strikingly practical character, and the author's enlarged general views of the nature and proper treatment of cancer. In the pages of Dr. Walshe, the disease is no longer the local, external, or *surgical* malady which it has been too much the fashion to consider it; but a true constitutional, general, *medical* disease, of which the local manifestation, particularly on the surface, is a comparatively small and unimportant feature. A very large part of the treatise is devoted to the disease as it affects internal organs, the diagnosis of which is here put in a much clearer light than heretofore. The strongest proof we can adduce of the constitutional nature of cancer is the fact, incontrovertibly made out by Dr. Walshe, that extirpation or ablation of the local affection by the knife, should only be had recourse to in an infinitely small proportion of cases.

Before introducing to the reader the subject of *compression*, which is the more immediate object of this notice, we cannot resist the pleasure of quoting two other short passages, which will give a little insight into some of the original and ingenious views of the author.

### *Dr. Walshe's views of the Nature and Progress of Cancer.*

"I had scarcely commenced the study of adventitious products, before I became convinced that much of the obscurity pervading the subject arose less from its nature, than from the erroneous manner in which its investigation had been conducted. I found observers had overlooked the fact, that the higher orders of these products were real existences developed within existences, and possessed of two distinct modes of life: a life, subject to its variations of health and disease, irrespective of the organism in which they had taken birth; and a life influenced by the conditions of the various structures and functions of that organism. I saw that phenomena, accessory and contingent, were confounded with others necessary and essential; and that, as a natural consequence, misapprehension of many pathological relations had followed. Desirous of removing this source of unsound doctrine, as it affects the most important of adventitious growths, Cancer, I have separately considered (on the plan habitually employed in my Lectures) the healthy and diseased conditions of vitality of that product." (Preface, p. i.)

"The following is the view which, I conceive, may be taken of the nature and sequence of the phenomena of the disease. A certain constitutional state exists, and may continue to exist for a variable period without giving functional evidence of its presence, although the blood and solids of the body are specially modified. In consequence of local injury, or otherwise, exudation takes place; upon that exuadation the constitutional state has impressed special attributes and tendencies, (p. 50;) among these attributes ranks an intrinsic power of vegetation. This vegetating faculty of the exudation reacts on the system by constantly draining it of a portion of its nutrient materials,—the progeny feeds upon the parent organism, and the first phasis of evolution is accomplished. But the natural tissues have been so modified in properties by the constitutional state, that they are incapable of resisting the encroachments of the vegetating exudation, and hence become the seat of atrophous, ulcerative, and other modes of destruction. Discharges of various kinds now still further drain the system of its fluids, and impair its vital energies; and the second phasis is established. Meanwhile secondary alteration of the blood is effected; this fluid becomes the vehicle for the circulation through the system of elements possessed of a germinating force,—these stagnate, are deposited, and new local vegetations spring into life and activity. The same series of phenomena is again and again gone through; until the system, drained of its reparative fluids in feeding exudations and supplying discharges, exhausted of almost every drop of pure blood through the influence of secondary cancerous impregnation, paralysed in its nervous energies by physical anguish and deficiency of pabulum, sinks in the struggle against the superior powers of the new existences it has created,—and in death is closed the third phasis of the disease." (pp. 189-90.)

### *Of Compression in the Treatment of Cancer, more especially by means of Dr. Arnott's Apparatus.*

"In the year 1809 Mr. Samuel Young conceived and acted upon the idea that the continued nutrition of scirrhous tumours might be completely prevented, and the absorption of existing substance insured, by submitting them to methodic compression. The results of the practice, as made public by himself, have been condensed as follows by Dr. A. L. J. Bayle. The number of cases given is nineteen; of these, seventeen relate to cancer of the breast, two to ulcers of the cheek and upper lip. Twelve cases terminated by cure; five were considerably benefited; the two cutaneous ulcers improved somewhat. The majority of the tumours were hard, irregular, tuberculated, and the seat of lancinating pain; six of them were ulcerated, and discharged ichorous pus. Even in the worst cases the tumour diminished in size, but the patients fell victims to the diathesis.

"In consequence of Mr. Young's announcements, the plan was tried at the Middlesex Hospital, and a committee appointed to report upon the results. The conclusion of the report, drawn up by Sir Charles Bell, was, that compression could not be regarded as a 'specific' cure, and had no claims to notice *except for its power of alleviating pain.* But, as was justly rejoined by Mr. Young, much of the want of success described may have arisen from defective management of the plan; no details of the cases are given. The spirit in which Sir Charles Bell judged, may be inferred from the allusion to the mode of treatment as a specific; a character with which Mr. Young never sought in the remotest degree to invest it; it would in truth be just as wise, observes the latter, to speak of the pad of a hernial truss as a specific against strangulation, as to assign the character to compression in cancer.

"The testimony of Mr. Travers (loc. cit. p. 306) is favorable to the practice. He has known tumours, such as those already described (p. 206), 'gradually reduced, and at length absorbed by equal and persevering compression, as by strips of soap and adhesive plaster, or, what is better, by an elastic roller passed many times round the chest, with layers of the Amadou smoothly interposed between the turns of the roller.' M. Recamier has employed compression upon a very large scale, and the more important part of his results is as follows: 'Of one hundred cancerous patients, sixteen appeared to be incurable, and underwent only a palliative treatment; thirty were completely cured by compression alone, and twenty-one derived considerable benefit from it; fifteen were radically cured by extirpation alone, or chiefly by extirpation and pressure combined, and six by compression and cauterization; in the twelve remaining cases the disease resisted all the means employed.' MM. Blizard and Masson have published three cases, and M. Carron du Villards three others,—in all of which irregular nodular scirrhi, the seats of lancinating pain, &c., were removed by compression. Dr. A. L. J. Bayle (loc. cit.) gives, as the general results in 127 recorded cases, 71 cures, 26 instances of improvement, 30 of total failure. These results, the most favorable on the whole that can be adduced in favour of any mode of treatment, bear scrutiny of the severest kind. It is no doubt true, that, in some of the cases alleged to be cancerous, neither of the anatomical species of that affection existed; but it is on the other hand perfectly unquestionable that many of the absorbed growths were not only actually scirrhous, but had already become the seat of ulceration, when submitted to compression.

"Difference of opinion has existed as to the best mode of applying compression. M. Recamier employs perfectly smooth disks of agaric, laid over each other, and retained *in situ* by a roller, as the compressing materials. M. Bégin sometimes substitutes a laminated plate of lead, modelled to the tumour, and surmounted with a pyramid of graduated compresses. This application (which is far from a novel one) frequently becomes painful, and cannot be endured. M. R. recommends a renewal of the apparatus every day, or every second day: M. Bégin thinks it better to change only when the bandages grow loose; and prefers, in consequence of this view, an elastic corset, capable of accommodating itself to the decreasing size of the part, as the compressing agent, wherever circumstances admit of its use. But all contrivances of these kinds are ineffectual, for various reasons: in the first place, they exercise unequal and irregular pressure on the tumour; in the second, they confine the movements of the chest to a degree varying with the amount of constriction; in the third, the force employed is not directed against the diseased mass alone, but wasted in great measure upon the healthy parts; and, in the fourth, while the difficulty of applying the apparatus effectually is extreme, it invariably loosens and becomes more or less disarranged within a short period after its application. Besides all this, the least unevenness in the material lying next the diseased structures renders the compression unbearable, from the pain it produces. These are the chief reasons, doubtless, which have hitherto prevented compression from taking its ground as a general system of treatment of various external cancers.

### *Dr. Arnott's Plan.*

"The fertile ingenuity of Dr. Neil Arnott (already so successfully and so variously employed in devising mechanical means of relieving human suffering) has triumphed over these difficulties. Dr. Arnott has invented a method of applying compression, which, while it is free from all the objections mentioned, is philosophical in principle, and possessed of peculiar practical excellences. His apparatus consists of a spring, an air-cushion supported by a flat resisting frame or shield, a pad, and two belts. The spring, which is of steel, is the compressing agent,—its strength being varied with the amount of pressure it may be desirable to obtain. The shield varying in shape somewhat with the circumstances of particular cases, is generally slightly convex on the external surface, of circular or oval outline, and formed of a rim of strong wire, connected at two opposite points by a flat piece of iron, which serves for the support of the spring, screws, &c., the whole being covered with jean. To the rim of this shield is sewn a sort of conical cap of soft linen, designed to receive the air-cushion, to keep it *constantly slack*, and prevent it from slipping about when applied. The air-cushion thus kept slack, fashioned into a sort of double night-cap, lying in apposition with the inner surface of the shield, and sufficiently filled with air to prevent the latter from pressing directly on the part, receives within it the tumour to be compressed. One end of the spring is attached by screws to the external surface of the frame, and the other end to a solid but soft pad, placed wherever the counter-pressure is to be made. The straps are used to keep the apparatus steadily fixed. Let us suppose that the breast is the region to which the instrument is to be applied; the position of its various parts will appear, as they are represented in the figures. The spring may either be passed over the shoulder or round the waist; the latter mode of application suits best, when the tumour is seated towards the external border of the breast, and inclined to slip towards the axilla.

"The mechanical advantages of this mode of compression are, that the movements of the thorax are not interfered with; that the amount of pressure may be regulated to a nicety; that the whole morbid mass undergoes constant, equable, and uniform pressure; that the part is protected from external injury, (a point of serious importance;) and that, unless in a very few exceptional cases, the apparatus may (either with the shoulder or waist-spring) be very easily arranged. It is necessary that the amount of pressure should be low at first (say 2lbs.,) especially in the cases of nervous, irritable people,—in fact, that the instrument should rather supply a support for, than exercise pressure on, the part; that the entire morbid structure (as well as any connected loose soft parts, which might be injured by accidental pressure of the rim of the shield,) should be included within the cushion; and that in all cases there be a distinct thickness of air-cushion between the shield and the skin.

"The effects produced by pressure are removal of existing adhesions, total cessation of pain, disappearance of swelling in the communicating lymphatic glands, gradual reduction of bulky masses to small, hard, flat patches or rounded nodules (which appear to be, both locally and generally, perfectly innocuous,) and in the most favorable cases total removal of the morbid production. The relief of pain afforded by the instrument is, without exaggeration, almost marvellous; this effect is insured by the peculiar softness and other properties of the air-cushion, the medium through which the pressure of the spring is transmitted to the surface. Females unable to obtain sleep even from enormous doses of laudanum cease instantaneously to suffer on its application; and sleep thenceforth, as though they were perfectly free from the disease.

"There are certain conditions which either interfere altogether with the use of the instrument, or reduce it to a merely palliative agent. These conditions are, more particularly, excessive bulk of the new growth, and such localizations of this structure as place any portion of it beyond the reach of pressure. Persons of irritable skin and temperament, and prone to become œdematous or anasarcous, are with some difficulty manageable. Less is to be expected in cases of encephaloid cancer than of the other species, and in cases of infiltrated than of tuberous accumulation. If the morbid mass be extensively softened, ulcerated, or in a state of fungous vegetation, palliation is all that can be fairly hoped for. Adhesion to the skin is of untoward influence. As a means of controlling and averting hemorrhage, the slack air-cushion pressure is of high utility.

"Pressure, on Dr. Arnott's system, is applicable to cancerous tumours in every situation where a bony or other solid support exists behind the growth, and where a point for counter-pressure can be had. The mamma, the limbs, the surface of the thorax or cranium generally, are the seats in which the mode of treatment is most readily applicable. I see no reason why cancer of the testicle might not be treated thus; and gentle pressure on this plan deserves a trial in certain cases of cancer of internal parts (it would surely relieve pain), provided the general functional relations of those parts do not interfere (and they will often not do so) with the adoption of such pressure.

"The system of pressure now described, useful as it is independently of all other treatment, may be rendered more efficacious by the association of other external means and internal remedies. The following case exemplifies the power of such combinations.

### *Case of Cancer by Dr. Walshe.*

"I was requested (March 3d, 1843) by Mr. Langley to see a lady affected with scirrhus of the breast, which it was proposed to remove with the knife. Exactly five months and a half ago was attracted by slight pain to the right breast and found there a lump about the size of a small hazel-nut, not tender to the touch, unattended with soreness or discoloration of the skin; it increased but little in size till the last six weeks, within which time it has enlarged to its present bulk; suffers scarcely any pain in the tumour itself, but has lancinating pain above the nipple in the indurated part of the gland to be presently described. In its general outline the right breast is double as large as the left (it has always been somewhat the fuller of the two;) the subcutaneous veins more visible than on the opposite side; at the axillary border of the gland is an excessively hard, solid, defined, rather moveable tumour; the finger may almost be slipped behind this, but at its inner edge it is continuous with another indurated mass, obviously a portion of the mammary gland itself in a state of infiltration; the tumour is finely knotted on the surface, the infiltrated part somewhat more largely so; besides this, the substance of the gland is indurated and knotty, especially above the nipple; the nipple is less prominent than on the healthy side, but is not actually drawn in; the areola is unaffected; there are no adhesions of the skin or alterations of its texture; no enlarged glands in the axilla, but slight thickening and hardening of some of the absorbent vessels leading thither; no discharge of blood ever from the nipple; when the tumour examined, not painful, but a short while after became so. Measurements: whole breast 4¾ inches broad, 3¼ inches vertically; tumour 1¾ inches broad, 1½ inches vertically. The soft parts about are flaccid and yielding. I recommended the following pill: ℞. Arsenici ioduret. gr.j. extract. conii ℈ij. in pill. xvj. dividend: j. bis die s. Light nutritious diet; moderate walking exercise. March 8. Applied the slack air-cushion (diameter of bag 6¼ inches, pressure of spring 3¼ pounds;) the whole mass of the breast included, except about half an inch at the left superior angle, where merely cellular tissue. No annoyance of any kind experienced by the patient, except slight impediment to respiration, which ceased in a few minutes. March 10. Pain totally removed; size of general mass of breast somewhat diminished, but the tumour is only rendered more prominent and apparent by this. No inconvenience is experienced from the instrument except in the back; the patient being thin, the pad presses uncomfortably on the spine. March 12. Tumour appears very distinctly reduced in bulk, more elastic, less stony in feel, less pointedly knotty on surface, less sharply defined: all these changes are to a very small amount, but they are nevertheless positive. Catamenia for last two days. March 30. Tumour (which has been gradually decreasing) is now scarcely more than half the original size; some slight indication of absorbents with tenderness of the skin, and slight redness; nothing in axilla; (instrument much disturbed yesterday in the carriage, and edge pressing against skin.)

"From this period until the middle of August the progress of things was slow and interrupted; twice, at the menstrual period, the tumour enlarged slightly, without however becoming painful. I gradually increased the force of the spring (which had always been carried over the shoulder) to six pounds, and diminished the diameter of the bag 3½ inches,—by which a great increase of pressure was obtained; and, at the date named, the tumour was about one third only of its original size, had become freely moveable under the skin, and the general knottiness and hardness of the gland had almost disappeared; patient had lost altogether the headaches which used to torment her. I now lost sight of the patient (first through my own, and then her absence from town) till the middle of November. During all this period the instrument had been more or less neglected, and not applied at all for the last month; the use of the pills had also been interrupted. November 23, 1843. The tumour is now at least half as large again as when I first saw it; it is more painful than ever, and has reacquired all its original characters; it is, however, still non-adherent. I reapplied the cushion, of the diameter first used, and within a week a favorable change had taken place; the tumour continued thenceforth to diminish in size, until it was reduced to the size of a hazel-nut. This little nodule appearing immoveable, I (at the close of January, 1844) directed the iodide of lead ointment to be smeared on the part twice daily, the pressure being at the same time continued. The effect was almost immediate, so much so that the patient, after the lapse of a fortnight, requested to be allowed to use the inunction without the pressure: the impulse to absorption had been given; the tumour steadily decreased in size, and had totally disappeared at the close of April, 1844. I have within the last few days (August, 1845) examined the breast, and found it in every respect like its fellow, *without a vestige of tumour or induration of any kind.*

"Here then was a tumour, which (though it had so far not given rise to any of the more terrible evils appertaining to cancer) yet possessed in the clearest and best defined manner the sum of characters assigned by universal experience to growths pursuing the common course of that disease; and this tumour disappeared completely under the persevering use of the means described. Had the growth been removed with the knife, the chances (as will fully appear in the sequel) are extremely strong that, within the present period, the disease would have reappeared, and perhaps destroyed its victim. But from the very perfection of success in this and similar cases an objection may arise in some minds. It may be urged that, as in such cases a mass, composed of indefinitely vegetating cells, is removed from its original site by absorption, the displaced cells may in some new abode germinate and flourish. But the objection is a fallacious one. The absorption effected in such cases must, on physiological grounds, be considered of the kind I have termed *unproductive* (see p. 105;) and clinical experience, so far as it has yet gone, corroborates, in the non-reproduction of tumours thus dispersed, the justness of the physiological principle." (pp. 207-14.)

In the original work, Dr. Arnott's apparatus and its mode of application are illustrated by a woodcut. And we may here observe, that the volume also contains one beautiful copper-plate, exhibiting the general phenomena of the pathology of cancer, and several other woodcuts devoted to the same object.

